# The co-management of tuberculosis-diabetes co-morbidities in Indonesia under the National Tuberculosis Control Program: results from a cross-sectional study from 2017 to 2019

**DOI:** 10.1186/s12889-022-13017-y

**Published:** 2022-04-08

**Authors:** Weixi Jiang, Trimawartinah, Fauziah Mauly Rahman, Adik Wibowo, Adhi Sanjaya, Permata Imani Ima Silitonga, Shenglan Tang, Qian Long

**Affiliations:** 1 Global Health Research Center, Duke Kunshan University, No. 8 Duke Avenue, Kunshan, 215316 Jiangsu China; 2 Department of Public Health, University of Muhammadiyah Prof DR Hamka, South Jakarta, Indonesia; 3 Global Health Initiative Faculty of Public Health, Universitas Indonesia, Depok, West Java Indonesia; 4 SingHealth Duke-NUS Global Health Institute, Singapore, Singapore; 5 Duke Global Health Institute, Duke University, Durham, NC USA

**Keywords:** Tuberculosis, Diabetes, Health policy, Health system

## Abstract

**Background:**

Indonesia suffers from a high burden of tuberculosis (TB) and diabetes (DM). The government initiated national TB-DM co-management activities under the National TB Control Program in 2017. This study investigates the detection and treatment outcomes of TB-DM in Jakarta after implementing these activities, and identifies the main factors associated with these outcomes.

**Methods:**

A cross-sectional study was conducted using TB registry data in two districts of Jakarta, East Jakarta (low-income) and South Jakarta (high-income). A 5-step cascade analysis was used: diagnosed TB patients; TB patients tested for DM; diagnosed TB-DM patients; and patients received and completed TB treatment/cured. We conducted descriptive analyses to understand the characteristics of TB and TB-DM patients, and used a two-level mixed-effect logistic regression to explore factors associated with having a DM test and completing TB treatment/being cured.

**Results:**

Over the study period (2017–2019) 50.8% of the new pulmonary TB patients aged over 15 were tested for DM. The percentage increased from 41.7% in 2017–2018 to 60.1% in 2019. Of the TB patients tested for DM, 20.8% were diagnosed with DM. Over 90% of the detected TB-DM patients received standard TB treatment, 86.3% of whom completed treatment/were cured. Patients in East Jakarta were more likely to be tested for DM and to complete standard TB treatment/be cured than patients in South Jakarta (*P* <  0.001). Bacteriologically positive TB patients were more likely to be tested for DM (OR = 1.37, 95% CIs 1.17,1.60). Patients diagnosed in sub-district level healthcare centers had a higher likelihood of being tested for DM than those in government and private hospitals (*P* <  0.05). Receiving DM treatment was associated with a higher likelihood of completing TB treatment/being cured (OR = 1.82, 95% CIs 1.20, 2.77).

**Conclusions:**

TB-DM case detection significantly improved in 2019 after introducing TB-DM co-management activities in Jakarta, while gaps in TB-DM co-management existed between bacteriologically positive and clinically diagnosed TB patients, and across different types of health facilities. Collaboration between TB and DM departments should be strengthened, and more resources need to be mobilized to further improve the co-management of TB-DM in Indonesia.

## Background

Tuberculosis (TB) and Diabetes mellitus (DM) are major global health challenges. TB is responsible for over one million deaths each year, and DM affects 463 million adults globally; a rate that has more than tripled over the past two decades [1, 2]. A double burden of TB and DM is of growing concern, especially in low- and middle-income countries (LMICs) with high burdens of TB [3]. Studies have found that TB and DM can each increase the incidence of the other [4], and DM can triple the risk of developing TB [5]. Results from bi-directional TB and DM screening all over the world have found large variations of TB prevalence in DM and DM prevalence in TB, ranging from less than 2% to over 35% for both rates. This variation is due to the wide variety in prevalence of each disease [6]. A systematic review of studies in South Asia suggested that DM prevalence among TB patients is higher in countries with a high TB burden [7]. Moreover, TB patients with DM are more likely to have adverse treatment outcomes such as relapse or even death, and potentially show a higher risk of developing multi-drug resistant TB [7–9]. Uncontrolled diabetes (plasma HbA1C level ≥ 7.0%) has been identified in studies as a risk factor for poor TB treatment outcomes, or even treatment failure [10, 11]. In 2011, the World Health Organization (WHO) and the International Union against Tuberculosis and Lung Disease released the “Collaborative framework for care and control of tuberculosis and diabetes”. This framework recognized the close correlation between TB and DM, and called for increased efforts to establish collaboration in TB-DM co-management [12]. The detection and treatment of TB-DM cases are crucial to reaching the end TB target of Sustainable Development Goals (SDGs) [13]. Achieving this target is a critical challenge, especially for LMICs with a high burden of TB and rising prevalence of DM.

The technical guidelines for the detection and clinical management of TB-DM co-morbidities have been widely covered in the current literature. Previous studies have discussed the technical validity and feasibility of TB-DM bi-directional screening. A two-stage approach including random plasma glucose (RPG) screening plus glycosylated hemoglobin A1C testing for RPG > 6.1 mm/L has been verified as an accurate approach to detect DM in TB patients in a multi-site large study [14]; the first step of which includes the use of fasting blood glucose and urine dipstick [6, 14]. The WHO recommends a five-point questionnaire on TB symptoms to screen for suspected TB cases before administering diagnostic tests such as sputum smear test, culture tests or X-rays [15]. Although recent studies on TB and DM in LMICs supported the need for DM screening among all TB patients or at least high-risk patients [7, 11, 16–21]; studies on the feasibility and impact of TB screening among DM patients have showed mixed-results even in countries with a high TB burden like India [22–26]. Nevertheless, the WHO still recommends active screening of TB among DM patients in countries with high TB prevalence (over 100/100000) [27]. The clinical management of concurrent TB-DM cases requires more effort, as TB patients with DM are more susceptible to the toxicity of TB drugs and to drug-drug reaction, which could result in poor treatment adherence [28]. Glycemic levels also need continuous monitoring during TB treatment to avoid adverse clinical outcomes.

Indonesia suffers from a high burden of TB and DM. According to a 2020 WHO report, Indonesia accounted for the second largest number (8.5%) of global TB incidence [2]. Over 6% of adults aged 20–79 in Indonesia have DM, a significant number given the large population of Indonesia [1]. A recent study estimated an age-standardized DM prevalence rate of 11.3% among Pulmonary TB patients in Indonesia [20], another study from 2013 to 2016 found that over 13% of DM cases in Indonesia ever had TB or were diagnosed as TB [29]. In 2015, the Indonesian Ministry of Health issued a Consensus on the Management of Tuberculosis and Diabetes Mellitus (TB-DM) to support comprehensive TB-DM co-management in health care facilities. This consensus included bi-directional screening algorithms, diagnosis pathways and referral requirements for TB-DM cases, and was intended as a reference for health workers on TB-DM management services in all primary health care (PHC) facilities in Indonesia [30]. In 2016, the first official statement of collaborative TB-DM co-management was included in National TB Control Program (NTP) and these co-management activities were initiated across Indonesia in 2017 [31]. According to the practice guideline of these TB-DM co-management activities, blood sugar test should be carried out immediately after the TB diagnosis is made in PHCs. The healthcare workers in the TB department of PHCs are responsible for reporting the test result and manage the TB-DM patients. TB information system has also been updated to record DM diagnosis and treatment among TB patients. Since its implementation, there has been little evaluation on the success of the TB-DM co-management program in real-life settings. This study aims to examine the case-detection and treatment outcomes of TB-DM patients in Jakarta, Indonesia after implementing the co-management activities, and to identify factors associated with the detection and treatment outcomes of TB-DM co-morbidities. This study will also provide new evidence regarding the impact of the TB-DM co-management program on overall TB-DM care, as well as implications for implementing such programs in similar resource-limited settings in other LMICs.

## Methods

### Study setting

This study was conducted in two districts of Jakarta, East Jakarta and South Jakarta. Jakarta is the capital of Indonesia with one administrative division and five municipalities. South Jakarta is a higher income residential region and East Jakarta is a lower income industrial region. In 2020, the population of South Jakarta was 2,226,812 with a density of 14,430 people per km2, while East Jakarta had a population of 3,037,139 with 16,624 people per km2. In 2017, the total monthly average expenditure on food and non-food items was USD 203 for residents in South Jakarta and USD 139 in East Jakarta, indicating the better socio-economic status of residents in South Jakarta [32].

### Study design

A cross-sectional study was conducted using data from the TB registry.

### Patient and public involvement statement

Patients or the public were not involved in the design, or conduct, or reporting, or dissemination plans of our research.

### Data collection

Data was retrieved from the Regional Health Office SITT (Sistem Informasi Tuberkulosis Terpadu) TB surveillance database for East and South Jakarta. Our time frame began with the dates of which DM information began to be recorded in the registry. This included the 4th quarter of 2017 in East Jakarta and 2018 in South Jakarta, and ended in December 2019 for both regions. The dataset included: (1) TB patients’ demographic information such as age, sex, place of origin and current address; (2) TB diagnostic and treatment information including the health facility, TB tests conducted, treatment length and treatment outcome; and (3) DM test outcome and DM treatment status. Patients with new pulmonary TB infections, aged over 15 years, and who were drug-sensitive were included in our analysis. TB patients with HIV were excluded.

### Data analysis

We used cascade analysis to understand the case detection and TB treatment of TB-DM patient for both districts and each district separately following the five steps: (1) Diagnosed TB patients; (2) TB patients ever tested for DM; (3) Diagnosed TB-DM patients; (4)TB-DM patients who received standard TB treatment; and (5) TB-DM patients who completed standard TB treatment/were cured [33]. A cured patient was defined when a bacteriological positive pulmonary TB patient had a (bacteriological) negative result in one test preceding the end of treatment as well as the final test at the end of treatment. TB treatment was considered completed when patients finished the required treatment period, had a negative sputum test during treatment but had no bacteriological test records at the end of treatment. Both categories are considered successful treatment outcomes and were combined for analysis.

Descriptive analyses were conducted to compare the characteristics of TB and TB-DM patients., Given the correlation of patients within the same healthcare facility, we used a multivariate two-level random-intercept model to explore factors associated with the likelihood of a DM test being administrated to TB patients, and completing TB treatment or being cured. The fixed effect part of the model include the following variables available in the dataset: region, (East Jakarta/South Jakarta), year of registration (before 2019 or 2019), gender, age (15–44 or >/=45), type of diagnosis (bacteriologically confirmed/clinically confirmed), patients’ origin (in/outside the region of registration, type of health facility where the patient was reported (health center at sub- sub district level/health center at sub district Level/government hospital/ private hospital), and the status of DM treatment (used for modeling treatment outcomes only). The variation at the healthcare facility level was modeled as a random intercept. Single variate analysis was conducted for factors associated with receiving standard treatment, due to the high likelihood of this scenario. The SITT database has very few missing data regarding the variables for analysis in this study. For the analysis on the likelihood of a DM test being administrated to TB patients and the likelihood of receiving standard TB treatment for TB-DM patients, there were no missing data. As only 50 TB-DM patients (1.8%) had missing data regarding TB treatment outcomes, we simply excluded these patients in this step of analysis. The data was analyzed using Stata 16 (StataCorp, Texas, USA).

## Results

### Cascade analysis of TB-DM case detection and treatment

**Fig. 1:**
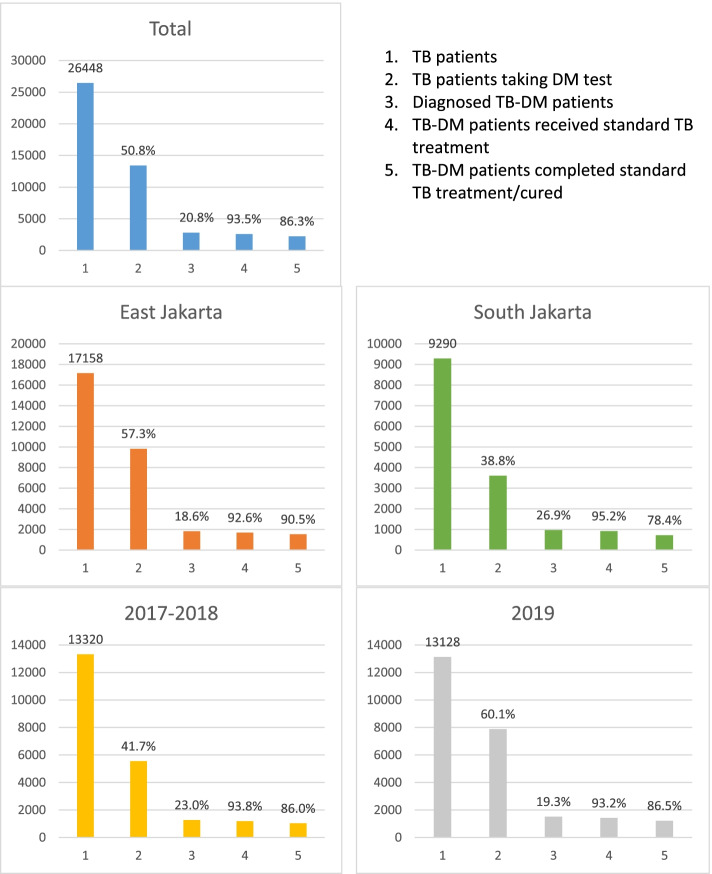
Case detection and TB treatment cascade for TB-DM patients in East and South Jakarta, 2017–2019

In total 26,448 patients were included in analysis. As shown in Fig. 1, 50.8% of these patients were tested for DM, 20.8% of whom were diagnosed TB with DM comorbidity. Of the diagnosed TB-DM patients, over 90% received standard TB treatment, 86.3% of whom completed treatment or were cured. A separate analysis of the two regions showed that the proportion of TB patients tested for DM was higher in East Jakarta (57.3%) than in South Jakarta (38.8%) (*P* <  0.001). The percentage of TB-DM patients who completed standard TB treatment/were cured reached 90.5% in East Jakarta, compared to only 78.4% in South Jakarta (*P* <  0.001).

In 2019, the percentage of TB patients ever tested for DM therefore increased from 41.7% in 2017–2018 to 60.1%, while the percentage of those diagnosed TB-DM patients among the tested decreased from 23.0 to 19.3%, a minor decrease compared with the increase in the proportion of patients tested. Meanwhile, the proportion of TB-DM patients initiating standard TB treatment remained around 93%, and treatment outcomes remained almost unchanged at about 86% in 2017–2018 and in 2019.

### Patient characteristics

Table 1 shows the characteristics of registered TB patients without DM diagnosis (TB-n-DM) and TB-DM patients in East and South Jakarta, as well as *P*-values of Chi-square tests. Patients in East Jakarta accounted for around 65% of both TB-n-DM and TB-DM cases, and around 60% of the TB-n-DM and TB-DM patients were male. The percentage of patients aged over 45 was higher among TB-DM patients (78.9%) than in patients without DM diagnosis (39.2%, *P* <  0.001). This finding is consistent with the fact that age is risk factor associated with TB-DM co-morbidity. Around 90% of the TB patients and 95% of the TB-DM patients had ever taken TB etiological tests, and the proportion of patients who had ever taken a rapid molecular test were higher among TB-DM patients (*P* <  0.001).

**Table 1:** Characteristics of TB patients and TB-DM patients in East and South Jakarta

	TB patients without DM diagnosis		TB-DM patients		*P*-value
	N	%	N	%	
Total	23,652	/	2796	/	NA
Region					
East Jakarta	15,332	64.8	1826	65.3	0.615
South Jakarta	8320	35.2	970	34.7	
Year					
2017* (Q4)	1746	7.4	133	4.8	< 0.001
2018	10,298	43.5	1143	40.9	
2019	11,688	49.1	1520	54.4	
Gender					
female	10,022	42.2	1147	41.0	0.175
male	13,630	57.8	1649	59.0	
Age					
15–44	14,380	60.8	589	21.1	< 0.001
> =45	9272	39.2	2207	78.9	
Patients’ origin (by ID card)					
in this region	19,648	83.1	2445	87.4	< 0.001
outside this region	4004	16.9	351	12.6	
TB treatment status					
received standard treatment	20,264	85.7	2613	93.5	< 0.001
non-standard	3388	14.3	183	6.5	
Diagnosis Type					
bacteriologically confirmed	13,176	55.7	2104	75.3	< 0.001
clinically diagnosed	10,476	44.3	692	24.7	
TB test taken					
Acid-Fast Bacillus (AFB) smear test	12,847	48.6	1440	51.5	
rapid molecular test	10,406	39.3	1593	56.9	< 0.001
culture test	453	1.7	73	2.7	
no test records	2756	10.4	135	4.8	
Treatment outcome					
complete treatment	11,489	48.6	968	34.6	< 0.001
cured	7931	33.5	1286	46.0	
default	1968	8.3	218	7.8	
transfer	973	4.1	129	4.6	
dead	470	2.0	75	2.7	
relapse	239	1.0	70	2.5	
not available data	582	2.5	50	1.8	
Type of facility where patients were reported					
health center at sub-district level	3385	14.3	560	20.0	< 0.001
health center at sub-sub district level	6585	27.8	926	33.1	
government hospital	8818	37.1	994	35.6	
private hospital	4864	20.6	316	11.3	
Receive DM Treatment					
metformin and others	/		2202	78.8	NA
insulin injection	/		172	6.2	
no DM treatment	/		422	15.1	

As for the treatment of TB and DM, 85.7% of the TB-n-DM patients and 93.5% TB-DM patients received standard TB treatment. In Indonesia, Metformin alone or in combination with other drugs were the most common oral therapies delivered at primary healthcare centers, and in our study 78.8% of the TB-DM patients were on such oral therapies, and 6.2% were receiving insulin injection.. It is also notable that the percentages of patients from the local area and patients with bacteriologically confirmed TB were higher among TB-DM patients than TB-n-DM patients (P <  0.001). Further analysis showed that 30.3% of bacteriologically confirmed TB patients tested for DM were diagnosed with DM, while the percentage was 18.8% for clinically diagnosed patients. In private hospitals TB patients may also be less likely to be referred for DM testing, or DM patients less likely to be referred for TB testing, as the percentage of cases reported from private hospitals was 20.6% for TB-n-DM patients but only 11.3% for TB-DM patients.

### Factors associated with the likelihood of ever having a DM test and completing TB treatment/being cured

Results of the LR test showed that both two-level mixed-effect logistic regression models fit significantly better than the simple logistic regression (*P* <  0.0001). The regression analysis showed that patients in East Jakarta were much more likely to have a DM test (OR = 5.32, 95% CIs 2.90, 9.79) after adjusting for other covariates, indicating strong regional differences in screening practices in Jakarta (Table 2). The increase in the percentage of TB patients tested for DM in 2019 was also significant after adjusting for other factors (OR = 4.23, 95% CIs 2.3, 7.78). Older patients (OR = 1.45, 95% CIs 1.31, 1.63) had a higher probability of getting a DM screening test, which also corresponds to the increasing risk of co-morbidity for the elderly. Bacteriologically confirmed patients were more likely to receive DM screening tests compared with clinically diagnosed patients (OR = 0.616, 95% CIs 0.580, 0.654). As for the types of medical facilities, patients who were diagnosed in sub-district level health centers were more likely to be tested for DM than in all other types of facilities including health centers at sub-sub district level, government hospitals and private hospitals (OR = 3.86, 95% CIs 1.20, 12.38). In general, both government and private hospitals may be less likely to perform DM tests than health centers (OR = 0.30, 95% CIs 0.12, 0.77 for government hospitals, OR = 0.07, 95% CIs 0.03, 0.17 for private hospitals).

**Table 2:** Factors associated with the administration of DM tests for TB patients

	OR	95% CIs	*P*-value
*N* = 26,448			
Region			
South Jakarta	ref.		
East Jakarta	5.32	2.90–9.79	< 0.001
Year			
before 2019	ref.		
2019 year	4.23	2.30–7.78	< 0.001
Gender			
male	ref.		
female	1.00	0.93–1.08	0.97
Age			
15–44	ref.		
≥ 45	1.45	1.31–1.62	< 0.001
Type of diagnosis			
clinically confirmed	ref.		
bacteriologically confirmed	1.37	1.17–1.60	< 0.001
Patients’ origin (by ID card)			
in this region	ref.		
outside this region	0.99	0.90–1.10	0.86
Type of health facility			
health Center at sub- sub district Level	ref		
health Center at sub district Level	3.86	1.20–12.38	0.023
government hospital	0.30	0.12–0.77	0.012
private hospital	0.07	0.03–0.17	< 0.001
Random Intercept			
healthcare facility	5.55	4.23–7.27	

When examining factors associated with the likelihood of receiving standard treatment, single-variate analysis found that type of diagnosis was the determining factor as almost all (179/183, 97.8%) diagnosed TB-DM patients who did not receive standard treatment had been clinically diagnosed (OR = 0.75, *P* < 0.001). Multivariate analysis (Table 3) shows that after adjusting for other factors, patients who received standard treatment in East Jakarta were more likely to complete such treatment or be cured (OR = 2.14, 95% CIs 2.90, 9.79). Receiving DM treatment was also associated with a higher likelihood of completing treatment/being cured (OR = 1.82. 95% CIs 1.20, 2.77). Patients aged over 45 may be less likely to complete treatment/be cured compared to younger patients, although this finding was not statistically significant (*P* = 0.059).

**Table 3:** Factors associated with completing treatment/being cured for TB-DM patients

	OR	95% CIs	*P*-value
*N* = 2563			
Region			
South Jakarta	ref.		
East Jakarta	2.14	2.90–9.79	< 0.001
Year			
before 2019	ref.		
2019 year	1.03	0.75–1.39	0.87
Gender			
male	ref.		
female	0.89	0.72–1.10	0.291
Age			
15–44	ref.		
>/=45	0.77	0.59–1.01	0.059
Type of diagnosis			
clinically confirmed	ref.		
bacteriologically confirmed	0.90	0.64–1.26	0.539
Patients’ origin (by ID card)			
in this region	ref.		
outside this region	0.96	0.69–1.33	0.807
Type of health facility			
health Center at sub- sub district Level	ref		
health Center at sub district Level	0.87	0.56–1.35	0.526
government hospital	0.93	0.57–1.52	0.786
private hospital	0.58	0.23–1.51	0.266
DM treatment status			
without DM therapy	ref.		
with DM therapy	1.82	1.20–2.77	0.005
Random Intercept			
healthcare facility	0.77	0.42–1.43	

## Discussion

### Summary of major findings

This study found that TB-DM case detection significantly increased in 2019 compared to the initial years (2017 and 2018) of implementation of the national TB-DM co-management activities in Jakarta. It is notable that the increased number of DM tests in 2019 still resulted in a rather high rate of diagnosed TB-DM cases among those tested (19.3%), suggesting the necessity and high efficacy of DM screening among TB patients. From our communication with collaborators, we also learned that there was a mass screening of both communicable and non-communicable diseases among staff in government and private companies which may contribute to the increase in TB-DM case detection. The initiation and completion of TB treatment, as well as the status of receiving DM treatment remained stable after program implementation. Patients who received DM treatment were associated with better TB treatment outcomes. These findings suggest the importance of continued TB and DM treatment, especially as cases are expected to increase, and indicate the need for additional efforts to improve treatment capacity for better -co-management of TB-DM cases.

### Factors associated with the detection and treatment of TB-DM cases

The study found significant differences in the likelihood of ever taking a DM test between bacteriologically confirmed and clinically diagnosed TB patients, as well as differences in receiving standard TB treatment for TB-DM patients. Considering that DM detection rate was higher among bacteriologically confirmed TB patients, DM screening could be made a priority for bacteriologically confirmed patients in resource-limited settings. Nevertheless, the fact that 40% of bacteriologically confirmed patients were untested for DM in East and South Jakarta also strongly suggests the need to expand DM screening. The proportion of bacteriologically confirmed diagnosis among TB patients also needs to be improved in Indonesia to facilitate more standardized practices of TB-DM management. Studies in several high TB burden countries have found that rapid molecular tests such as GeneXpert showed improved performance in detecting *Mycobacterium tuberculosis*, especially for cases without typical symptoms, and recommended the use of such technology in similar settings [34–36]. Though GeneXpert has been included in the national guidelines of TB diagnosis in Indonesia, the percentage of TB patients receiving a rapid molecular test was less than 50, and 42.2% of the TB patients were diagnosed without bacteriological evidence in our study. In terms of such a low coverage, the use of rapid molecular test technology such as GeneXpert could be further promoted in Indonesia.

Our study also found that TB patients in government and private hospitals had a lower likelihood of being tested for DM compared to those in healthcare centers, suggesting sub-optimal implementation of TB-DM co-management at the hospital level under NTP. In Indonesia, only the primary healthcare centers are under the direct leadership of the NTP [37]. Previous studies have found sub-standard TB management practices among hospitals and private practitioners in Indonesia [38, 39]. Despite a technical guideline on the management of TB-DM patients in advanced referral health facilities (mainly hospitals) issued by the Ministry of Health in 2015, the performance of hospitals in case detection still seems worse than in primary healthcare centers. One study in an Indonesian hospital revealed gaps between guideline requirements and implementation regarding TB-DM co-management, and found that insufficient human resources contributed to these shortcomings [31]. Another study on referral practices of private practitioners in Indonesia found that receiving information on standard practices as well as supervision from district program officers were associated more better performance [40]. In light of these findings, strengthening health worker training and improving supervision, especially for hospitals and private practitioners is necessary to improve TB-DM co-management.

### Suggestions to further improve TB-DM co-management in Indonesia and other LMICs

Coordination between TB and NCD control programs needs to be improved to strengthen TB-DM co-management. Though the WHO has also called for the establishment of a formal collaboration between the NCD prevention and TB control programs in TB-DM co-management [12], TB and DM service integration has been especially challenging as in the past they have been individual vertical programs delivered under different divisions of the health department with little collaboration [41–44]. In Indonesia, only NTP issued guidelines on TB-DM co-management while the NCD department has not. The responsibilities of reporting and recording TB-DM cases fell solely on the TB department, while the NCD department did not report TB-DM case and just transferred them to TB department when they discovered them in the screening test. Other LMICs face similar challenges. For example, in India and Bangladesh the TB-DM co-management also fall under the national TB control programs [45, 46], and they suffer from insufficient human resources as well as the lack of a registration system for TB-DM cases in the DM department [23]. In addition to case detection and reporting, clinical management of TB-DM also needs efforts from DM department to develop optimal regimen which takes into account patients’ glycemic control needs and their higher likelihood of developing adverse drug effects than non-DM TB patients [47, 48]. As revealed in our study, the proportion of DM screening among TB patients is still relatively low, and there are disparities in TB-DM co-management across regions, by types of health facilities and patient types (bacterially confirmed or clinically diagnosed cases). In light of these challenges, more domestic and international financial resources need to be mobilized to improve test capacity as well as support and supervise healthcare workers in TB-DM patient management. However, currently there are huge gaps in the funding for TB and NCD control in general in Indonesia. According to WHO the funding gap for TB in Indonesia is 74% [49]. Funding for NCDs are also far from insufficient in most LMICs to support long-term and affordable care for DM patients [50, 51]. Besides, the soloed funding structures of TB and NCD programs hinders collaboration between the programs [44]. The COVID-19 pandemic has caused huge burden on the healthcare system and impacted health service delivery in many countries. Integrated care would improve efficiency of health service delivery, particularly regarding the deployment of healthcare human resources in resource-limited settings. Therefore, both domestic and international funds should be raised and directed towards promoting coordination between NTP and NCD control programs to mobilize and allocate health resources effectively and improve overall efficiency.

### Limitations and implications for future studies

This study has several limitations. One major limitation is that all data were retrieved from TB registry, as there were no TB-DM reports in the NCD registry. Additionally, we could not identify how many TB-DM cases were detected through DM screening among TB patients, or TB screening among DM patients from TB registry data. Nor could we estimate the efficiency of screening in either direction. Besides, we could not exclude the possibility that healthcare workers in the PHC filled in a previous DM test result, as the date of DM testing was not recorded. Nevertheless, we did observe a significant increase in the percentage of TB patients tested for DM in 2019, which was in line with the recommendation of DM screening among TB patients in the clinical practice guideline. Furthermore, we did not have data on the changes in DM prevalence in Jakarta from 2017 to 2019. However, as we observed a 33% increase in diagnosed TB-DM cases from 2018 to 2019, such a significant increase is not likely a result of change in DM prevalence. Another restriction is that the study is limited to data from Jakarta, the capital city, and therefore we are unable to analyze the implementation of TB-DM co-management activities in other regions of the country. Future studies may consider using data covering more regions across Indonesia. For example, data could be obtained from primary healthcare centers and hospitals in both urban and rural areas to analyze the implementation of bi-directional screening and co-management of TB-DM. Potential interventions that consolidate the efforts of TB and DM departments should be explored to further improve the co-management of TB-DM.

## Conclusions

TB-DM case detection improved in 2019 after the introduction of the TB-DM co-management activities in Jakarta, and the initiation and completion of TB treatment remained stable during the implementation period. Receiving DM treatment was associated with better TB treatment outcomes for TB-DM patients. Nevertheless, gaps in TB-DM co-management still exist across regions and between bacteriologically confirmed and clinically diagnosed patients. Government and private hospitals were also less likely to follow guidelines to test for DM among TB patients than primary healthcare centers. Collaboration mechanisms between national TB programs and NCD programs should be established and strengthened. Finally, more resources need to be mobilized to further improve the capacity of TB-DM co-management in Indonesia and likely other LMICs.

## Data Availability

The data that support the findings of this study are available from the Regional Health Office of East Jakarta and South Jakarta, but restrictions apply to the availability of these data, which were used under license for the current study, and so are not publicly available. Data are however available from the authors upon reasonable request and with permission of the the Regional Health Office of East Jakarta and South Jakarta.

## Abbreviations

TB: Tuberculosis
DM: Diabetes mellitus
LMICs: Low- and middle-income countries
WHO: World Health Organization
SDGs: Sustainable Development Goals
RPG: Random plasma glucose
NTP: National TB Control Program
